# Intraoperative Heparin Resistance and Internal Carotid Artery Occlusion After Andexanet Alfa Reversal During Endovascular Treatment of a Ruptured Cerebral Aneurysm: A Case Report

**DOI:** 10.7759/cureus.105094

**Published:** 2026-03-12

**Authors:** Shin Kawamura, Jo Matsuzaki, Naoki Yamamoto, Toru Yamagata, Misao Nishikawa

**Affiliations:** 1 Department of Neurosurgery, Moriguchi Ikuno Memorial Hospital, Osaka, JPN; 2 Department of Stroke Neurology and Neuroendovascular Surgery, Moriguchi Ikuno Memorial Hospital, Osaka, JPN

**Keywords:** andexanet alfa, direct factor xa inhibitors, heparin resistance, large-vessel occlusions, subarachnoid hemorrhage

## Abstract

A 56-year-old woman receiving oral edoxaban (30 mg/day) was transferred to our hospital with impaired consciousness and headache. Head computed tomography revealed a subarachnoid hemorrhage and a 7-mm aneurysm located at the junction of the left internal carotid artery-posterior communicating artery. Andexanet alfa was administered 11.5 hours after the patient’s last dose of edoxaban to reduce the potential rebleeding risk before aneurysm treatment. During coil embolization, acute occlusion of the left internal carotid artery was observed. Mechanical thrombectomy was subsequently performed, achieving successful reperfusion. Postoperative magnetic resonance imaging revealed an acute cerebral infarction in the watershed areas. The patient was discharged 48 days after onset with a modified Rankin Scale score of 0. Although impaired anticoagulation response and thrombotic events have been reported following andexanet alfa administration, acute large-vessel occlusion can also occur in this setting. Careful consideration of risk-benefit balance is warranted when performing neuroendovascular procedures requiring systemic heparinization.

## Introduction

Direct factor Xa inhibitors are widely used for the prevention of embolic events in patients with nonvalvular atrial fibrillation and for the treatment and prophylaxis of venous thromboembolism; however, major bleeding remains a clinically significant complication [1]. Andexanet alfa is currently the only specific agent approved for the “reversal of anticoagulation in patients treated with direct factor Xa inhibitors when they experience life-threatening or uncontrolled bleeding” [1]. In the ANNEXA-4 trial, andexanet alfa demonstrated effective hemostasis in patients with major bleeding, although thrombotic events were observed in 10.4% of patients within 30 days [1,2].

In patients with subarachnoid hemorrhage (SAH), early hemostatic stabilization is crucial because rebleeding is associated with poor neurological outcomes. In this context, reversal of anticoagulation may be considered when clinically indicated. Ruptured intracranial aneurysms are typically treated either by microsurgical clipping or by endovascular coil embolization. Endovascular treatment generally requires systemic heparinization to prevent thromboembolic complications during catheter manipulation. Thus, clinicians may face a clinical dilemma: reversal of anticoagulation may reduce the risk of rebleeding, whereas systemic heparinization during endovascular treatment is required to prevent thromboembolic complications. Neuroendovascular procedures themselves carry a risk of thromboembolic complications, and potential interactions between anticoagulation reversal and intraoperative anticoagulation have been discussed [3]. The safety profile of andexanet alfa in patients undergoing urgent neuroendovascular procedures remains incompletely defined. Therefore, the decision to administer andexanet alfa in patients with SAH who require urgent endovascular treatment remains complex and must be individualized.

Here, we report a case of intraoperative large-vessel occlusion observed during endovascular treatment after preprocedural administration of andexanet alfa for a ruptured cerebral aneurysm.

## Case presentation

A 56-year-old woman presented with impaired consciousness and a severe headache. Her past medical history included mitral stenosis and regurgitation, for which she had undergone bioprosthetic mitral valve replacement with left atrial appendage plication four years earlier. Postoperatively, an abnormal structure was noted on the replaced valve, and edoxaban (30 mg/day) was prescribed. Her medical history also included hypertension, but no known coagulation disorders. Her medications included edoxaban 30 mg/day, vonoprazan 20 mg/day, torasemide 8 mg/day, irbesartan 10 mg/day, amlodipine 10 mg/day, febuxostat 10 mg/day, and bisoprolol 1.25 mg/day. She had quit smoking four years prior and consumed approximately 1,500 mL of beer and 1,500 mL of Japanese spirits daily.

On the day of presentation, she took edoxaban at 09:00. At 19:30, she developed a sudden, severe headache, collapsed, and lost consciousness. Emergency medical services were called. Upon arrival, her vital signs were as follows: blood pressure 153/108 mmHg, pulse 92 bpm, temperature 36.5 °C, and SpO₂ 96% on a 5 L/minute face mask. On neurological examination, she was initially assessed as Glasgow Coma Scale (GCS) 4, improving to 9 upon hospital arrival. Pupils were equal and reactive (3.0/3.0 mm), and no focal deficits such as limb paresis were observed.

Laboratory findings on admission showed no marked abnormalities in platelet count, prothrombin time international normalized ratio (PT-INR), or activated partial thromboplastin time (APTT). Renal function was within normal limits.

**Figure 1:**
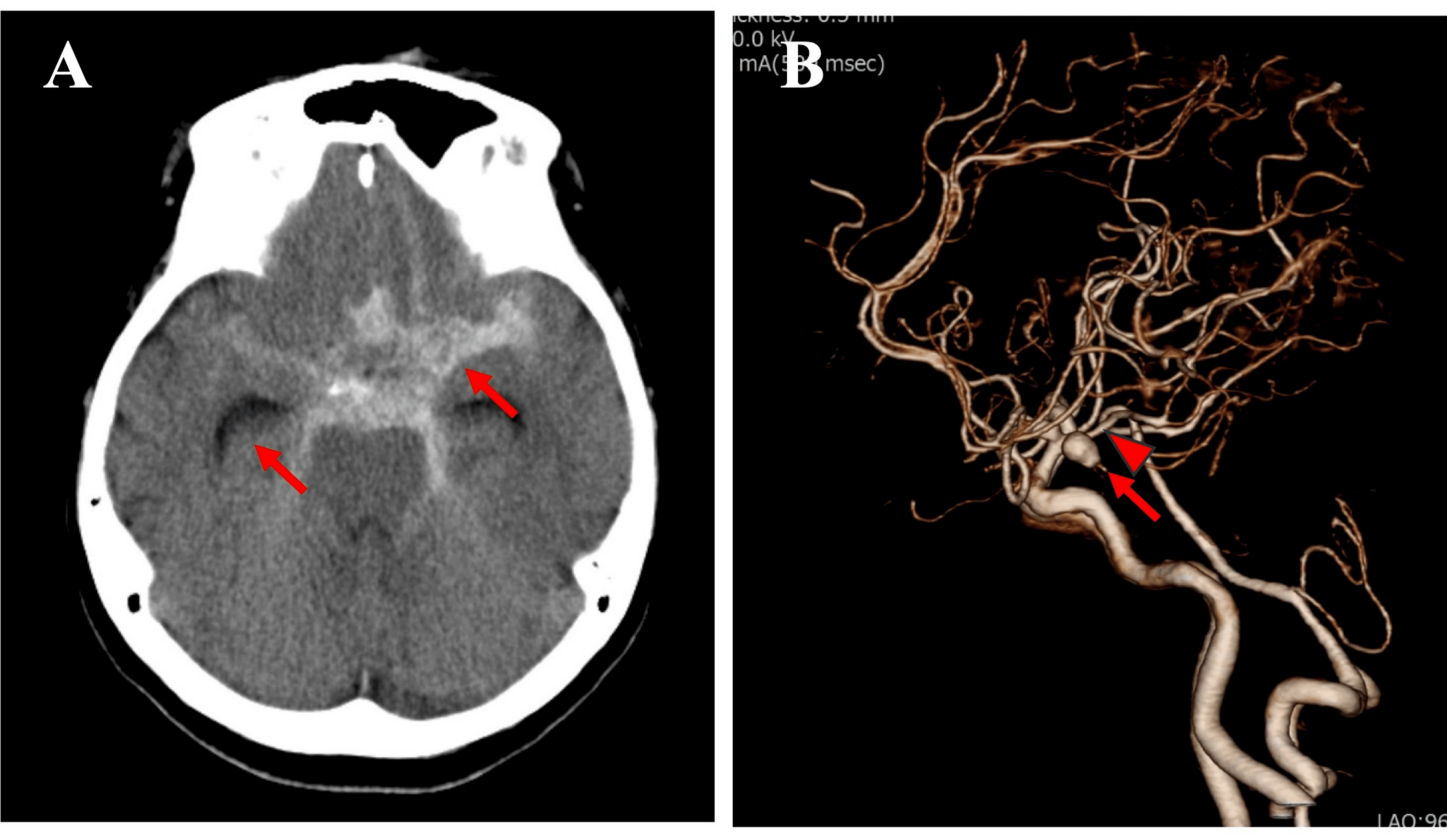
Neuroradiological findings on admission. (A) Non-contrast head computed tomography (CT) showing subarachnoid hemorrhage with ventricular enlargement (red arrows). (B) CT angiography demonstrating a 7-mm aneurysm at the left internal carotid artery-posterior communicating artery junction (red arrow). The left posterior cerebral artery was of the fetal type (red arrowhead).

Non-contrast enhanced head computed tomography (CT) revealed SAH predominantly distributed from the prepontine cistern to the suprasellar cistern and extending into the left Sylvian fissure, accompanied by acute hydrocephalus (Figure 1A). CT angiography demonstrated a 7-mm aneurysm in the left internal carotid-posterior communicating (IC-PC) artery. The left posterior cerebral artery was of the fetal type (Figure 1B). Evaluation of the aortic arch showed a bovine arch and an aberrant right subclavian artery.

A diagnosis of SAH secondary to rupture of a left IC-PC aneurysm was established (World Federation of Neurosurgical Societies grade II; Hunt and Kosnik grade III; Fisher group 3). Coil embolization was planned. Because the SAH occurred while the patient was taking edoxaban and approximately 11.5 hours had elapsed since the last dose, andexanet alfa was administered to reduce the potential risk of rebleeding before aneurysm treatment. Emergency coil embolization was performed later the same day.

**Figure 2:**
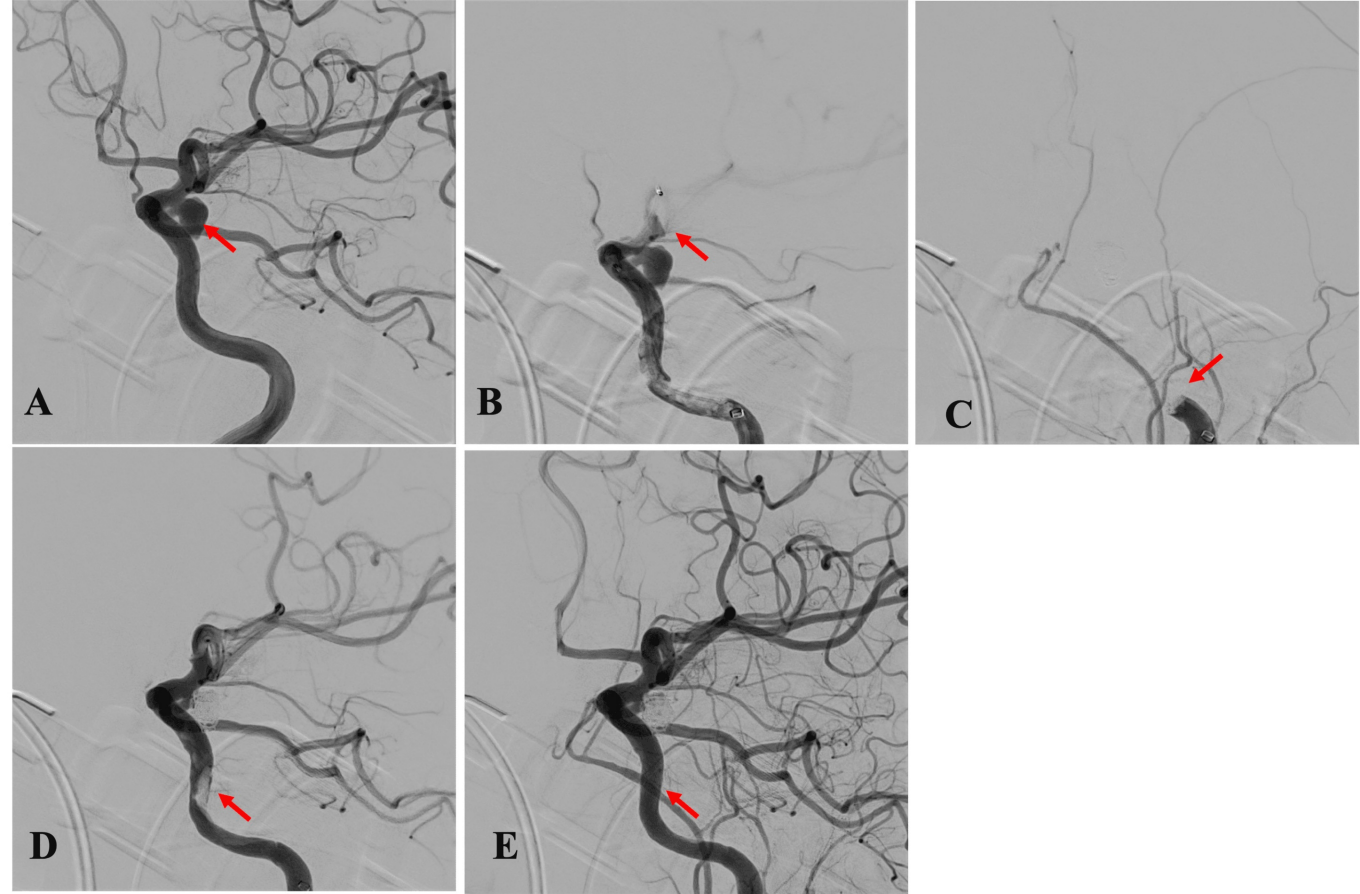
Neuroangiographical findings during emergency coil embolization. (A) A working view of the left internal carotid arteriography (I-CAG) for coil embolization revealed a 7-mm aneurysm at the left internal carotid artery-posterior communicating artery junction (red arrow). (B) Left I-CAG performed 1.5 hours after the start of the procedure demonstrated complete occlusion of the anterior and middle cerebral arteries (red arrow), along with a thrombus-like filling defect in the supraclinoid segment of the internal carotid artery (ICA). (C) Post-coil embolization angiography showed complete occlusion of the left ICA (red arrow). (D) Angiography after the first pass revealed a residual thrombus in the ICA proximal to the aneurysm (red arrow). (E) During a second pass, the combined technique was performed. Final angiography revealed modified Thrombolysis in Cerebral Infarction grade 3 recanalization (red arrow) and modified Raymond-Roy classification class II.

Under general anesthesia, a right femoral approach was employed. An 8F Optimo EPD Flex (Tokai Medical, Aichi, Japan) guiding catheter was positioned proximally in the left internal carotid artery (ICA). Digital subtraction angiography demonstrated a left IC-PC aneurysm measuring 7.3 × 5.3 × 5.1 mm with a 3.5-mm neck (Figure 2A). The initial activated clotting time (ACT) was 108 seconds, and 3,000 units of intravenous heparin were administered to maintain a target intraoperative ACT of 200-250 seconds. A 6F Navien (Medtronic, Minneapolis, MN) catheter was advanced coaxially with the 8F Optimo and positioned at the clinoid segment of the ICA. A SHOURYU2 4 × 10 mm (Kaneka Medix, Tokyo, Japan) balloon was initially attempted to be placed in the parent artery using a CHIKAI standard 14 (Asahi Intec, Aichi, Japan); however, advancing the CHIKAI standard 14 into the distal artery proved difficult and time-consuming. Approximately 15 minutes after the initial heparin bolus, the ACT remained 135 seconds; thus, an additional 1,000 units of heparin were administered. After successful placement of the SHOURYU2 balloon in the parent artery, the ACT remained 132 seconds (15 minutes after the additional heparin), prompting administration of an additional 2,000 units of heparin. The procedural timeline and anticoagulation response are summarized in Table 1. 

**Table 1:** Procedural timeline and anticoagulation response.

Time from last edoxaban dose	Event
0 hour (09:00)	Last dose of edoxaban
10.5 hours (19:30)	Onset of subarachnoid hemorrhage
11.5 hours	Andexanet alfa administered
13.5 hours	Endovascular procedure started
+15 minutes	Initial ACT 108 seconds → 3,000 U heparin administered
+30 minutes	ACT 135 seconds → additional 1,000 U heparin
+45 minutes	ACT 132 seconds → additional 2,000 U heparin (total 6,000 U)
+50 minutes	Thrombus detected in the supraclinoid internal carotid artery
+60 minutes	Successful recanalization after mechanical thrombectomy

Subsequent angiography via the Navien revealed a filling defect in the clinoid ICA suggestive of thrombus, accompanied by occlusion of the anterior cerebral artery and the middle cerebral artery (Figure 2B). An additional 5,000 units of heparin and edaravone were administered intravenously. The SHOURYU2 balloon was removed, and the procedure was converted to a simple coiling technique to prioritize hemostasis of the ruptured aneurysm. A Phenom 17 pre-shaped 90 (Medtronic) microcatheter was navigated over a Synchro Select Soft 14 (Stryker, Kalamazoo, MI) and positioned within the aneurysm. Three detachable coils were deployed, achieving a volume embolization ratio of 24%. Post-coil embolization angiography showed complete occlusion of the left ICA (Figure 2C). Although the angiographic occlusion status of the aneurysm could not be evaluated, intraoperative hemostasis was considered adequate. Coil embolization was completed, and the procedure continued with mechanical thrombectomy. A direct aspiration first-pass technique was performed using the Navien catheter, retrieving a large amount of red thrombus. Angiography after the first pass revealed residual thrombus in the ICA proximal to the aneurysm (Figure 2D). A combined technique using CELEGLIDE (Johnson & Johnson, New Brunswick, NJ) and Solitaire X 6 × 40 mm (Medtronic, Minneapolis, MN) stent retrievers was then employed to retrieve a moderate amount of red thrombus. Final angiography demonstrated modified Thrombolysis in Cerebral Infarction grade 3 recanalization and modified Raymond-Roy classification (mRRC) class II (Figure 2E). The femoral access site was closed with a Perclose ProStyle device (Abbott, Tokyo, Japan). 

**Figure 3:**
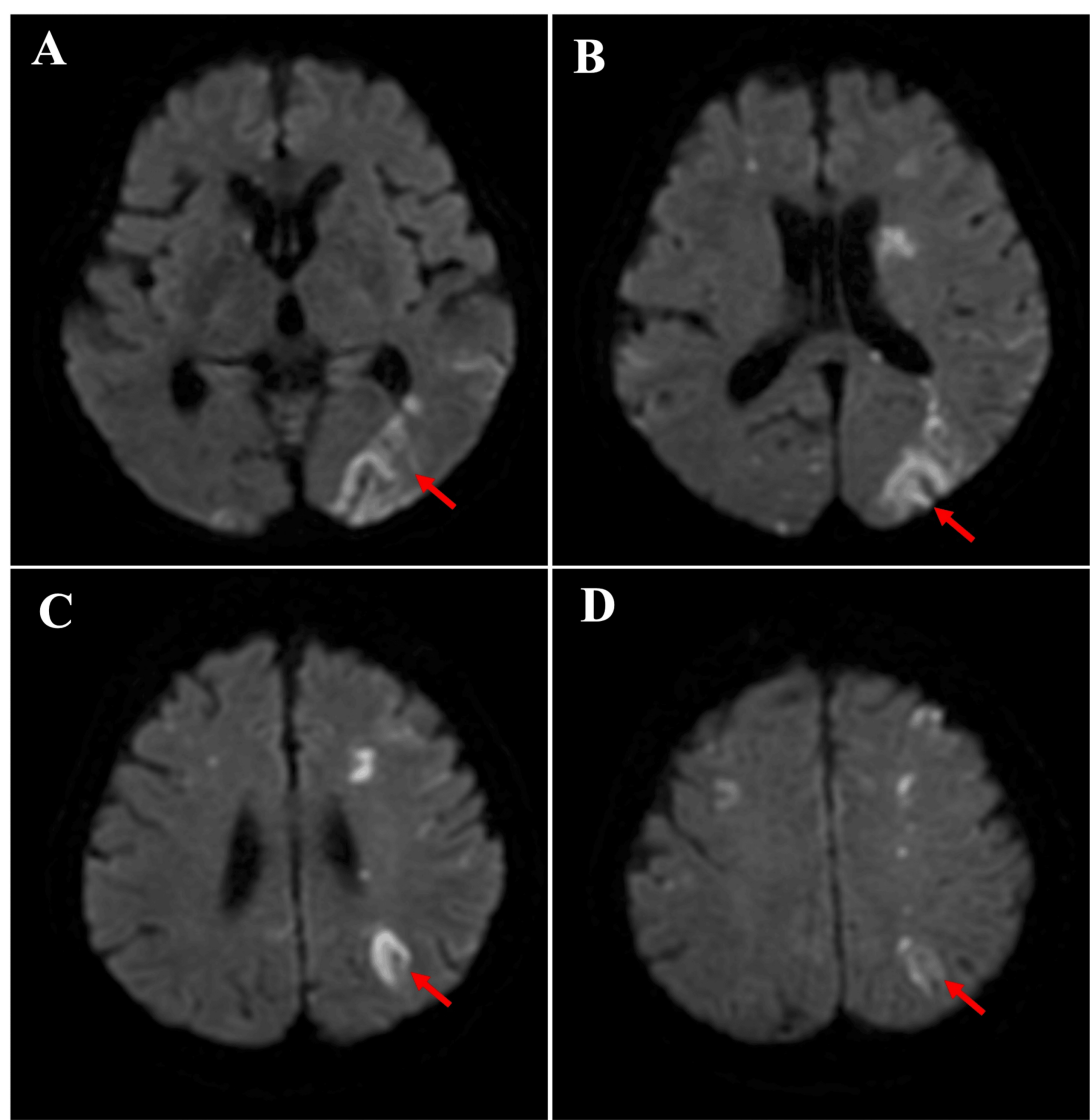
Neuroradiological findings after emergency coil embolization. Magnetic resonance imaging (MRI) of the head revealed acute cerebral infarctions in the anterior and posterior watershed areas and the deep internal border zones (red arrows).

Postoperatively, the patient exhibited mild right-sided hemiparesis. Head magnetic resonance imaging revealed acute cerebral infarctions in the anterior and posterior watershed areas and the deep internal border zone (Figure 3). The hemiparesis gradually improved. No complications, such as cerebral vasospasm or hydrocephalus, occurred, and the postoperative course remained stable. Angiography at one-month follow-up demonstrated complete aneurysm occlusion (mRRC class I). The patient was discharged 48 days after onset with a modified Rankin Scale score of 0.

## Discussion

**Figure 4:**
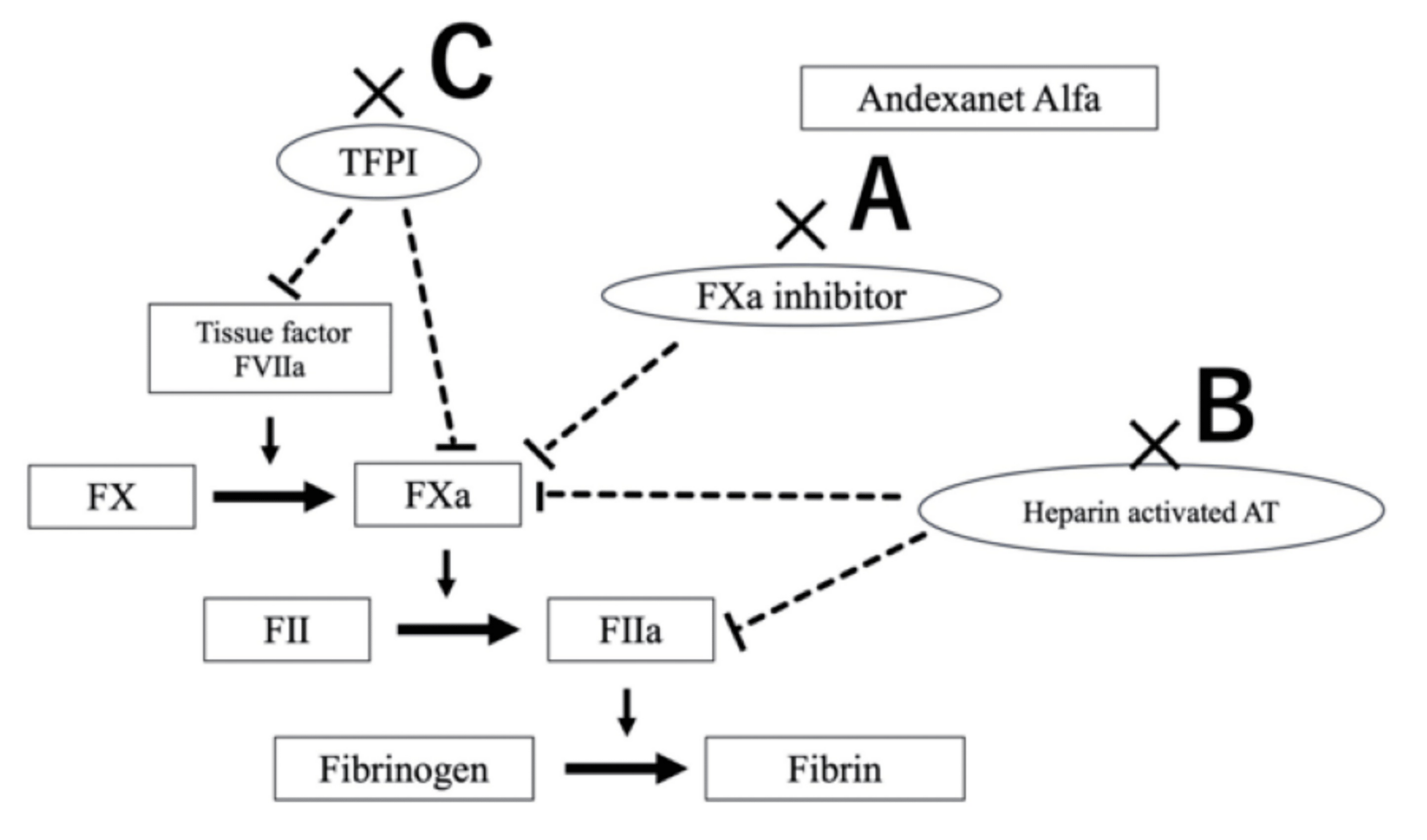
Schematic diagram of the blood coagulation system focusing on factor Xa inhibitors and andexanet alfa. (A) Andexanet alfa acts as a decoy receptor for factor Xa inhibitors, restoring endogenous factor Xa activity. (B) Potential attenuation of antithrombin (AT) mediated heparin anticoagulation. Andexanet alfa may interfere with the AT-heparin complex, reducing inhibition of thrombin and factor Xa and potentially resulting in an inadequate ACT response. (C) Possible reduction of tissue factor pathway inhibitor (TFPI) activity through binding to andexanet alfa, shifting the coagulation balance toward a prothrombotic state. Image credit: All authors.

Andexanet alfa is a genetically engineered, modified decoy of factor Xa. By acting as a decoy, andexanet alfa binds factor Xa inhibitors, preventing their interaction with endogenous factor Xa (Figure 4A). Consequently, endogenous factor Xa activity necessary for prothrombin activation is preserved, and coagulation function is restored. Through this pharmacologic mechanism, andexanet alfa has demonstrated effective hemostasis in patients receiving factor Xa inhibitors who present with acute major bleeding, as shown in the ANNEXA-4 trial [1]. However, thrombotic events occur with a certain frequency following andexanet alfa administration. In the ANNEXA-4 trial, 10.4% of 479 patients experienced thrombotic events within 30 days, including ischemic stroke in 4.6% [1]. A case of acute large-vessel occlusion immediately after andexanet alfa administration for thalamic hemorrhage, requiring recanalization therapy, has also been reported [2]. 

In this case, andexanet alfa was administered before aneurysm securing because rebleeding after SAH is strongly associated with poor neurological outcomes. Edoxaban has a reported half-life of approximately 10-14 hours, and although 11.5 hours had elapsed since the last dose, residual anti-factor Xa activity may still have been present. Conventional coagulation parameters were not markedly abnormal; however, routine laboratory tests do not reliably reflect the anticoagulant activity of factor Xa inhibitors. Therefore, reversal therapy was selected to reduce the potential risk of rebleeding before definitive treatment, based on clinical judgment at the time.

Heparin exerts its anticoagulant effect primarily by potentiating antithrombin (AT), which inhibits thrombin (factor IIa) and factor Xa. Adequate AT activity is therefore essential for heparin to prolong activated clotting time (ACT). In the present case, the expected ACT response following initial heparinization was not achieved. Despite administration of 3,000 units of intravenous unfractionated heparin and escalation to a cumulative dose of 6,000 units, the ACT remained subtherapeutic (135 and 132 seconds, respectively), below the target range of 200-250 seconds (Table 1). This inadequate ACT response persisted for approximately two hours after initial heparin administration and preceded the development of large-vessel occlusion. Andexanet alfa has been reported to interact with AT-heparin complexes, potentially attenuating the AT-mediated anticoagulant effect of heparin, resulting in functional heparin resistance (Figure 4B). Consequently, the European Medicines Agency recommends avoiding andexanet alfa in patients requiring heparinization [3], and a similar advisory has been issued domestically by the Japanese Society of Cardiovascular Anesthesiologists [4]. In addition, andexanet alfa may bind to tissue factor pathway inhibitor (TFPI), a key endogenous inhibitor of the tissue factor-factor VIIa complex and factor Xa. Reduction of TFPI activity may shift the coagulation balance toward a prothrombotic state (Figure 4C) [5]. Although causality cannot be definitively established in a single case, impairment of AT-mediated heparin activity together with potential TFPI inactivation may have contributed to inadequate intraoperative anticoagulation and subsequent thrombus formation. Beyond these pharmacologic mechanisms, endothelial irritation from catheter manipulation and the hypercoagulable state associated with acute SAH may also have contributed to thrombus formation.

Previous reports in the cardiothoracic field have demonstrated that andexanet alfa-associated heparin resistance can be successfully managed by supplementation of AT. Apostel et al. [6] reported prompt restoration of heparin responsiveness after AT administration in a patient with andexanet alfa-induced heparin resistance during cardiopulmonary bypass. However, evidence regarding such management strategies in the context of acute stroke care and neuroendovascular procedures remains limited. Accordingly, alternative management strategies could also be considered in such situations. These include AT supplementation to restore the anticoagulant effect of unfractionated heparin, or, when the clinical condition permits, delaying coil embolization until the following day after the prothrombotic effects of andexanet alfa have diminished. In addition, some authors have suggested the use of prothrombin complex concentrate as an alternative reversal strategy in patients with intracranial hemorrhage who may require procedures involving systemic heparinization [7].

Cardioembolic stroke accounts for 27.7% of all acute ischemic strokes [8]. The prevalence of atrial fibrillation increases with age, reaching 3.2% in individuals aged ≥ 80 years [9]. Thus, patients receiving direct factor Xa inhibitors are frequently encountered in daily clinical practice for cardiocerebrovascular disease. Such patients may also present with acute hemorrhagic conditions, including hypertensive intracerebral hemorrhage or ruptured aneurysm, for which andexanet alfa is recommended [10]. Although andexanet alfa is not formally contraindicated in patients with SAH, its indication is often challenging in clinical practice. Given the inherent thromboembolic risk of neuroendovascular procedures, administration of andexanet alfa should be carefully considered, as it may further increase this risk. In our case, timely revascularization achieved complete recanalization of the occluded vessel and was associated with a favorable neurological outcome. Nevertheless, this case underscores the critical considerations required when determining the indications for andexanet alfa.

## Conclusions

This case highlights the importance of carefully balancing the potential benefits and risks of andexanet alfa in patients with SAH receiving factor Xa inhibitors. When neuroendovascular treatment requiring systemic heparinization is planned, possible impairment of intraoperative anticoagulation and thrombotic complications should be taken into consideration. A multifactorial mechanism, including pharmacologic and procedural factors, may contribute to thrombus formation in this setting.
